# Prevention and treatment of psychosis in pregnant and/or postpartum women with known psychiatric illness – the state of the art of clinical practice guidelines

**DOI:** 10.1192/j.eurpsy.2025.364

**Published:** 2025-08-26

**Authors:** A. L. Falcão, B. S. Leal, I. M. Figueiredo, A. S. Lourenço, G. Soares, M. Nascimento, C. Oliveira, J. Reis

**Affiliations:** 1 Hospital Júlio de Matos, Unidade de Saúde Local de São José, Clínica 2; 2 Hospital Júlio de Matos, Unidade de Saúde Local de São José, Clínica 3, Lisboa, Portugal

## Abstract

**Introduction:**

Motherhood represents a challenge for all women, but it’s even more complex for those suffering from serious psychiatric illnesses such as Bipolar Disorder, Schizophrenia and Schizoaffective Disorder. The treatment of these women requires special care during the preconception, prenatal and postnatal phases, taking into account the risk of decompensation, the psychosocial factors involved and the difficult balance between the potential harm to the foetus and/or infant and the risks associated with not treating the mother. With the scarcity of randomised clinical trials and limited evidence, clinical practice guidelines become essential to determine the best therapeutic approaches to adopt.

**Objectives:**

To systematise the best evidence of care for pregnant and/or postpartum women with a history of psychotic illness.

**Methods:**

Systematic literature review.

**Results:**

Mental health management in women with severe psychiatric illness who want to become pregnant should involve shared decision-making and multidisciplinary counselling. In women of childbearing age who are diagnosed with such conditions, adequate awareness of the illness and the need for family planning is the first step towards effective and safe long-term treatment. In the prenatal period, it’s essential to monitor early signs of relapse, to psychoeducate about the need to stop comorbid consumption and to carry out additional foetal ultrasounds at specific times to rule out malformations in foetuses exposed to antipsychotics and lithium. In the postnatal period, the risk of relapse is especially high. Careful monitoring in the first month after birth and regular review thereafter are essential. When necessary, hospitalisation in mother-baby units is the *gold standard* treatment. Pharmacological treatment of pregnant and breastfeeding women should weigh up the risks associated with non-intervention and the potential adverse effects on the foetus and lactating infant. The choice of psychotropic drugs should taking into account the varying safety profiles; for example, typical antipsychotics can cause extrapyramidal symptoms or withdrawal syndrome in the newborn and atypical antipsychotics metabolic syndrome. Nevertheless, despite the quality of the evidence, antipsychotics appear to be generally safe in pregnancy and breastfeeding.

**Conclusions:**

The management of mental health care for this subpopulation must ensure that decisions are shared, follow-up is multidisciplinary, pre- and post-natal monitoring is individualised and pharmacological treatment is chosen based on the best balance between the needs of the mother and the safety of the foetus/infant.

**Disclosure of Interest:**

None Declared

